# New perspectives on evolutionary medicine: the relevance of microevolution for human health and disease

**DOI:** 10.1186/1741-7015-11-115

**Published:** 2013-04-29

**Authors:** Frank Jakobus Rühli, Maciej Henneberg

**Affiliations:** 1 Centre for Evolutionary Medicine, Institute of Anatomy, University of Zurich, Winterthurerstrasse 190, Zürich, 8057, Switzerland; 2 Biological Anthropology and Comparative Anatomy Unit, The University of Adelaide, Frome Road, Adelaide 5005, Australia

**Keywords:** anatomical variation, empirical, evolutionary medicine, microevolution, mortality, pathology, secular trends

## Abstract

Evolutionary medicine (EM) is a growing field focusing on the evolutionary basis of human diseases and their changes through time. To date, the majority of EM studies have used pure theories of hominin macroevolution to explain the present-day state of human health. Here, we propose a different approach by addressing more empirical and health-oriented research concerning past, current and future microevolutionary changes of human structure, functions and pathologies. Studying generation-to-generation changes of human morphology that occurred in historical times, and still occur in present-day populations under the forces of evolution, helps to explain medical conditions and warns clinicians that their current practices may influence future humans. Also, analyzing historic tissue specimens such as mummies is crucial in order to address the molecular evolution of pathogens, of the human genome, and their coadaptations.

## Definition, history of evolutionary medicine research and present situation

Evolutionary medicine (EM), or Darwinian medicine as it is sometimes called, investigates human disease vulnerability and disease etiologies (genetics, behavior, environment, pathogens, and so on) from an evolutionary perspective. EM is a biomedical scientific concept of increasing interest since the 1990s [1,2]. It has been the topic of several textbooks [3-5] and also recently a major scientific colloquium [6]. The intellectual beginnings of evolutionary medicine stemmed from the recognition that past evolutionary events can explain present-day conditions of the human body. Thus, by applying the concept of nature's evolution to human morphology, physiology and pathophysiology, a better understanding of the etiology of present-day human ailments can be achieved. Early applications of poorly understood Darwinian concepts to human biology led to eugenic theories [7,8]. However, EM (as we strongly emphasize here) does not deal with eugenic approaches. It espouses approaches to population biology that do not deal with individuals, but with intergenerational manifestations of biological processes that have no value attached to them.

Humans still evolve, in terms of anatomical structures and physiological processes as well as disease patterns and prevalence. The platonic, essentialist view that *Homo sapiens*, once formed, remains the same biological entity throughout the centuries is patently incorrect. Irrespective of the disparate views on the origin of humans held by adherents to different religions and scientific theories, changes in human genes and phenotypes from generation to generation do occur. Microevolutionary changes in human lineages during historical times are clearly understandable in the evolution of immunity to diseases, but also in the appearance of new metabolic processes such as lactose tolerance [9] or in the widespread acquisition of genetic variations in the capacity to process ethanol [10]. They have occurred in anatomical structures, too; such significant changes in morphological characteristics include: decrease in the robusticity of the musculoskeletal apparatus (gracilization) [11,12], weight and height [13], microcranialization and brachycephalization (reduction in braincase size and change of its shape) [14], reductions in the size and number of teeth [15] and spinal morphology alterations [16]. These alterations are all likely to be at least partially the result of structural reductions in response to technology diminishing the need for the use of physical strength and introducing extraoral food processing. Aside from genetic changes, such alterations may occur due to environmental changes such as a reduction of chewing effort in the processing of food, leading to a mechanically caused reduction in jaw size.

Microevolution is observable as a process of changes occurring in phenotypes of successive generations. These changes may result from changing, under the operation of forces of evolution, gene frequencies, or from adaptive phenotypic responses to changing living conditions. The changes of gene frequencies are a part of the general evolutionary process involving mating systems, drift, gene flow, mutations and selection. They can only occur through the process of reproduction that requires the genetic endowment of one generation to be passed on to the next generation. During that process frequencies of alleles or of genotypes can be altered leading to permanent alterations of immune responses, physiological processes and anatomical structures. Phenotypic adaptive responses are modifiable through alteration of living conditions during the lifespan of one generation, but only within the limits of genetically determined plasticity of individual responses to environmental stimuli.

Theoretically, the minimum time span required for microevolutionary change of the gene pool is that of two generations, while there is no constraint on the minimum time span for an adaptive phenotypic change. Since, due to a long human fertile lifespan of some 30 years, generations are widely overlapping in living populations, while living conditions in modern economies change rapidly, it is not always easy to distinguish between a truly evolutionary change and a phenotypic secular trend if the specific genetic determination of changing functions or structures is not known. Although classic descriptions of evolutionary processes refer to long time spans, there is no reason to expect that a change in gene frequencies may not occur during the time span of a century. The average age of parents at the time they produce offspring is approximately 20 to 40 years and thus on average three generations can be turned over during a century, each providing an opportunity for change of gene frequencies. This change may be rapid if a particular force of evolution operates strongly. For example, gene flow resulting from mass migration may profoundly alter the gene pool of a given geographic region within several decades.

Human microevolution has recently accelerated due to the rapid growth of human population numbers facilitated through cultural development and technologies [17]. Phenotypic manifestations of these changes are sometimes referred to as secular (derived from the Latin term *saeculum*, for 'a generation') or microevolutionary alterations. A distinction can be made between secular changes and microevolutionary alterations, based on their causes as explained earlier. Secular changes, such as increases in stature or weight are usually alterations of phenotypic expression of genetic potential without any changes in gene frequencies, while true microevolution involves change of gene frequencies, like in the case of accumulating mutations. Since for many morphological and physiological characters the exact mode of inheritance is not known, the distinction between phenotypic adaptive trends and true microevolution may be made by observing whether the magnitude of a particular change exceeds the range of adaptive phenotypic responses of the same genetic potential. If generation-to-generation changes exceed full phenotypic expression of the same genetic potential they can be considered microevolutionary ones, since they must reflect the changing genetic endowment of successive generations.

Most microevolutionary alterations have medical implications for individual patients (for example, knowledge of current anatomical variations for surgeons) as well as at the population level (for example, sociospecific public health measures). Studies of microevolutionary changes require time depths of at least a few generations, thus EM research specifically uses historic samples, where the time periods investigated extend over a number of centuries or even a few millennia. The value of such studies of ancient tissues has become more and more accepted even for clinical research, particularly as a crucial reservoir to study the evolution of infectious diseases [18-21].

The aim of this review is to highlight the potential of novel directions in EM empirical research for current and future biological and medical applications rather than discuss pure theoretical understanding of the origin of humans. Thus, it discusses present-day public health activities and biomedical practices from a perspective of future generations. Furthermore, the value of ancient tissue samples such as mummified bodies and archaeological bones and teeth to study recent evolution of human disease is addressed, as well as the possible impact of EM on academic curricula.

## Current EM research

Major fields of primarily non-clinical EM research to date have included aspects of demography [22], evolutionary genetics [23], sex [24], and socioanthropological issues [25]. The value of EM has been recognized particularly for clinical research [26,27]. Presently, EM concepts have been applied in clinical settings with a major focus on disease-provoking morphology, for example, of the human spine [28], on the changes in infectious diseases through time [3], explanations of psychiatric diseases such as depression, schizophrenia, anxiety disorders, and personality disorders [29-31], metabolic disorders such as iron deficiency [32] or nutrition-based pathological effects [33,34].

## Possible approaches in future EM studies

### Relaxed natural selection and microevolution of human morphology

One major field for future evolutionary research with a particular biomedical perspective is the study of alterations of natural selection, understood as differential reproductive success of carriers of different genes, and its impact on human morphology and pathology.

**Figure 1:**
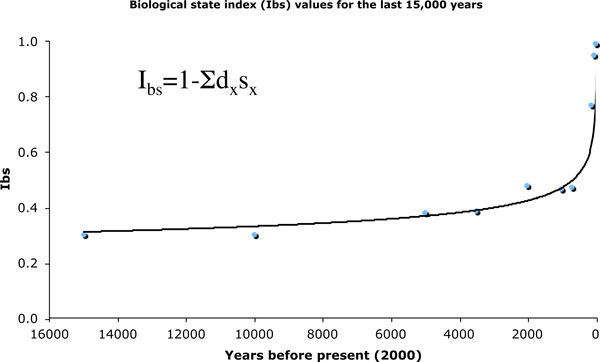
**Changes in the Biological State Index over the last 15,000 years of human evolution**. The index value is a probability that an average person will be able to fully participate in reproduction of the next generation. The lower the index value the greater the opportunity for natural selection. Labels in the formula are: d_x _= a fraction of dying people of age x; s_x _= reproductive value of a person of age x (for example, s_65 _= 0, while s_15 _= 1). For further explanation and data see [35,70].

Over the entire evolution of humankind, there was a very significant opportunity for the process of natural selection (Figure 1). It mostly occurred due to high levels of differential mortality that allowed less than half of individuals born to pass on their genes to next generations, eliminating the other half [35]. Until the mid-19th century infant and child mortality was so high that the survivorship to age 15 years was around 50% or somewhat less, even in countries presently considered to be 'developed' [36,37]. Although some deaths happened without a link to individual genetic endowment, many were linked to varying physical strength, levels of immunity, metabolic disorders (for example, type 1 diabetes, phenylketonuria), vision defects [38] and less common congenital defects. Differential fertility contributed much less to the overall opportunity for selection since there was little genetic variation in this characteristic [39]. This situation has changed drastically during the last approximately 150 years with the most welcome advent of sanitation and generally available medical treatments. The opportunity for natural selection through differential mortality has been so severely reduced that, at the end of the 20th century, more than 90% of newborns had an opportunity to fully participate in the reproduction of the next generation [36], while fertility became dependent on the conscious decisions of individuals and couples in both the sense of avoiding births and giving birth by infertile couples. For the first time in the evolution of humanity, the majority of natural selection pressures were relaxed to the apparent benefit of all of us. The increase in variability of heritable traits is a predictable outcome of such a relaxation of selection as its stabilizing effects are diminished [40]. This might not be true for psychiatric disorders, where social pressure may still influence reproductive success [31]. There is also evidence that, at least for some disorders, psychiatric disorders may be linked to allele variations that predispose to differential susceptibility and adverse effects in terms of developing a disorder [41]. At the genetic level, alleles do not have an absolute adaptive or maladaptive value, they assume it by interaction with the rest of the genome and the epigenetics determines their Darwinian fitness [42]. A gene producing pathological effects in the past (for example, predisposition to type I diabetes mellitus) may not be considered maladaptive in an environment where there is an effective treatment for diabetes.

**Figure 2:**
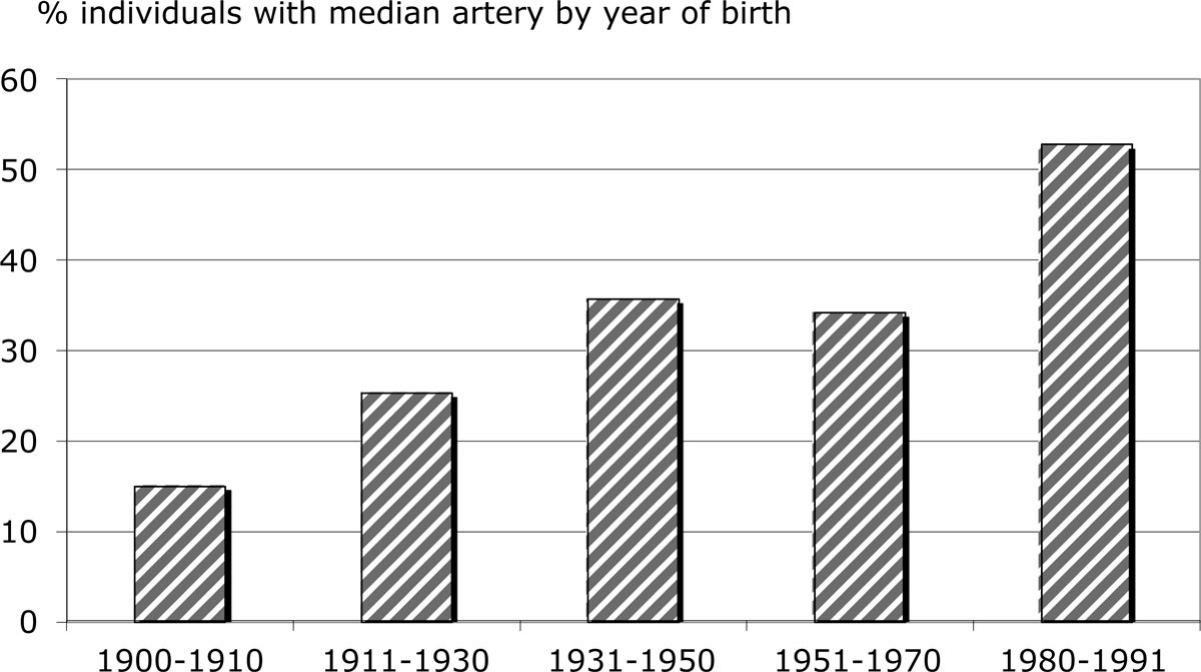
**Frequencies of individuals with median arteries of forearms by date of birth in a sample of 284 South African dissection cadavers**. Trend of increase in the incidence is significant (χ^2^_(1) _= 11.90, *P *<0.001 *z *= 3.94, *P *<0.0011 (*z *test for linear trends in proportions)). For further data see [71].

One can multiply such clinically relevant examples of relaxed natural selection. For example, an increase in the range of human biological variation has been already documented for a plethora of anatomical structures. Some 'anomalous' arteries have more than doubled their prevalence (for example, the median artery of the forearm is now present in around 30% of individuals in different populations, while at the beginning of the 20th century it was present in only around 10% of individuals [43]) (Figure 2), and the *thyroidea ima *branch of the aortic arch had disappeared completely by the end of the 20th century [44].

Climatic factors have been proposed to influence the altered prevalence of the internal thoracic artery [45]. In the skeletal system, opening of the sacral canal (*spina bifida occulta*) became more common in cohorts born in the second half of the 20th century than it was before [46], and tarsal coalitions appear more often in more modern times, too [47]. Skeletal pathologies such as ossification of the posterior longitudinal ligament of the spine have increased [48] as have diffuse idiopathic skeletal hyperostosis [49]. Many other rather short-term changes of body morphology, such as alterations in body dimensions and proportions (for example, body mass index, skeletal robustness or bone density) have also been shown. The widely reported secular increase in stature, which occurred with varying speeds (from 0 to over 150 mm per century [50]) in various populations, has affected body proportions since most of the stature increase, where it occurred, was due to the growth of the lower extremities [51].

Also, the current epidemic of obesity may in part result from increasing variation in the size of the body frame that reflects a greater variation in the size of the gastrointestinal tract [52] rather than just caloric imbalance. The range of variations of hormones regulating human appetite, for example, leptin and ghrelin and enzymes regulating carbohydrate and fat metabolism in past and present populations may differ, thus adding to the evolutionary explanation for part of the obesity problem. Although it can be argued that short-term changes in body height and body weight are not the result of changes in gene frequencies, but simply adaptive, non-heritable responses to changing living conditions, the ability of the human body to respond to such changes is a product of its earlier evolution. The response, especially in the case of increasing body weight, seems to be harmful and needs to be treated by interventions based on the understanding of human heritable adaptations to past diets, the so called thrifty genotype hypothesis debate (for example, modern diabetes-causing genes were advantageous in the past) [53]. The economic impact of such body shape alterations on 'biological standards of living' has been addressed previously [54]. Besides direct economic costs, obesity is linked to increased mortality and morbidity and thus any short-term alteration in obesity rates will have huge public health implications. Finally, it is also not clear if the entirety of body height increases that occurred during the 20th century are adaptive rather than of a genetic nature [55].

Regulation of postnatal growth and development has undergone a significant transformation during the last century. This change has become most obvious in the adolescent period [56]. Sexual maturity accelerated, while the rates of growth at puberty became much higher than before, resulting in problems in adolescence [56]. It remains to be determined to what extent acceleration of sexual maturation and increases in peak growth velocity are the result of alterations in socioeconomic conditions, and to what extent microevolution of human growth regulation has occurred. Studies of the occurrence of skeletal manifestations such as *hyperostosis frontalis interna*, may possibly further elucidate the recent evolution of the human endocrine system [57].

Other examples might be the alterations in prevalence and the etiology of metabolic syndrome, and the introduction of biologically active substances (for example, xenoestrogens or endocrine disruptors) into the food chain [58]. Finally, even within short time periods disorders of unknown cause, such as Paget's disease, can show a notable yet etiologically enigmatic alteration in prevalence [59]. Thus, recording these secular alterations is the very first step to explore possible environmental cofactors of such disorders. It is evident that our biological properties are changing even within very short historical time frames. More research elucidating what changes occur, with what intensity and to predict their biomedical consequences is needed, and should be a major future field of EM research.

### Lessons from paleopathology: Evolution of diseases and genomic studies

Of special importance to EM is the subdiscipline of paleopathology, which attempts to describe diseases in the past and to trace changes of those diseases in response to the historical development of humans, especially during the last several thousand years. From diagnoses of individual cases observed in ancient skeletons and in mummies, the discipline has evolved into palaeoepidemiological studies [60,61], even though meta-analytic standards known from clinical studies can hardly ever be met. Studies have discussed the impact of recent genetic sweeps such as the positive selection of Tay-Sachs disease-affected people versus tuberculosis [62]. Also, sex differences in genetic vulnerability to cancer or arteriosclerosis can be addressed by EM research.

Many attempts at explaining host-pathogen coevolution in relation to major infectious diseases such as leishmaniasis or plague have been made [63,64].

Epigenetics is another field for future EM research. Epigenetic factors mediating gene expression such as early-in-life stress ('fetal programming') would be one such example. Poor intrauterine conditions are predictive for somatic and psychiatric disorders, including maternal adversity [65]. Since it has been suggested that micro-RNA is linked with human pathologies such as cancer, molecular evolutionary studies may solve certain etiological enigmas. Another example, the lively and still continuing debate about the origin of syphilis [66,67], has stimulated closer scrutiny of pathogens, the study of their impact on the health of populations, systems of public health and ways of handling the recent resurgence of treatment resistant forms of the disease. For genomic studies in particular, new technological advances will allow more sensitive and specified research.

### Outlook

Medically oriented empirical research with an evolutionary focus may help to redesign public health policies and public awareness of science. A 'morphological anomaly' may become more frequent or even 'normal' in a given population and, thus, it should be no reason for concern for a particular individual. This needs to be realized and communicated accordingly (for example, by general practitioners to their patients). Accepting variation as normal is an important issue in clinical medicine.

To summarize, human biological traits still evolve. We are not simply 'stone age bodies in a modern world', but we are both at the same time adapted and adapting; biological compromises in a rapidly changing environment, with the latter also being full of coevolving pathogens. Therefore, future clinical studies in EM should focus particularly on the genomic evolution of bacterial and viral diseases and responses in the evolution of human immune system. For the latter, DNA viruses are easier to extract than RNA viruses. Issues such as viral pandemics or evolution of strain-dependent virulence can be explored by using a temporal and thus historical perspective. As highlighted previously [68], the imminent conflict of our short-term and long-term evolutionary genetic endowment is etiologically linked to the major causes of death in first-world countries such as cardiovascular or oncological disease. Thus, any progress in fighting disease based on evolutionary insights would be most welcome in the medical community, as well as in the general community. With the prospect of improved ancient DNA and proteomic analyses, we are now only at the edge of an entirely new era that will allow us to unravel the mysteries of human disease evolution. Furthermore, the incorporation of principles of human evolution and its forces into the knowledge of future medical practitioners is needed. A general practitioner may not directly heal a patient using only EM principles, but without any evolutionary knowledge he/she certainly will not be able to provide the best, individualized diagnosis, medicosocial advice and prescribe optimal personal treatment [69]. The same is true for any biomedical researcher; not applying EM principles may restrict the true scientific impact and applicability of a particular research result. Thus, the introduction of EM topics into medical (and science) curricula is recommended.

## Abbreviations

EM: evolutionary medicine.

## Competing interests

FJR has received research funding from the Mäxi Foundation. No other relevant conflicts of interest exist. MH has no conflicts of interest.

## Authors' contributions

FJR conceived the idea for this paper and discussed it with MH. MH produced the first draft, which was substantially expanded and modified by FJR. Both authors contributed examples from their earlier studies and incorporated them into the text. Both authors edited the text and approved the final manuscript.

## Pre-publication history

The pre-publication history for this paper can be accessed here:

http://www.biomedcentral.com/1741-7015/11/115/prepub
